# ECG-GraphNet: Advanced arrhythmia classification based on graph convolutional networks

**DOI:** 10.1016/j.hroo.2025.05.012

**Published:** 2025-05-19

**Authors:** Myeonghun Lee, Jiwoo Lim, JinKook Kim

**Affiliations:** 1 HUINNO Co., Ltd., Seoul, Republic of Korea; 2 School of Systems Biomedical Science, Soongsil University, Seoul, Republic of Korea; 3 Department of Biomedical Engineering, Ulsan National Institute of Science and Technology, Ulsan, Republic of Korea; 4 Department of Industrial Management Engineering, Korea University, Seoul, Republic of Korea

**Keywords:** Electrocardiogram, Cardiovascular disease, Arrhythmia, Machine learning, Deep learning, Graph convolutional network

## Abstract

**Background:**

Deep learning has significantly improved medical diagnostics, particularly in electrocardiogram (ECG) analysis, yet accurate classification of arrhythmias remains challenging.

**Objective:**

We propose Electrocardiogram Graph Convolutional Network (ECG-GraphNet), a graph convolutional network designed to classify arrhythmias into 3 types: normal (N), supraventricular ectopic (S), and ventricular ectopic (V) beats.

**Methods:**

ECG-GraphNet utilizes a novel graph representation of ECG data in which the P wave, QRS complex, and T wave are modeled as individual nodes. A unique QRS-centered weighted average pooling method is employed to enhance beat-specific feature extraction. We systematically explored various aspects including node features, edge definitions, a data augmentation method, and architecture configuration to determine the optimal model design. Experiments were conducted on 10-second ECG recordings from 328 patients using a single-lead device.

**Results:**

The optimized ECG-GraphNet achieved a Macro F1 score of 88.61% in 5-fold cross-validation. Scalability experiments further demonstrated its robustness, with Macro F1 scores of 85.21% and 87.03% across diverse ECG patterns and sizes.

**Conclusion:**

Our novel approach and comprehensive analysis underscore the potential advantages of ECG-GraphNet in clinical diagnosis and monitoring.

**Key Findings**

▪ We have developed ECG-GraphNet, which transforms P-QRS-T segments into graph nodes with dynamic edge relationships. This novel graph structure precisely captures both temporal and morphological features of cardiac signals, overcoming the limitations of traditional neural networks in processing variable-length ECG data.
▪ We introduce a QRS-centered pooling method specifically designed for ECG graphs that effectively preserves critical beat-specific information. This innovation significantly improves the detection of subtle beat-to-beat variations crucial for arrhythmia classification.
▪ Through extensive experimentation, we have developed an optimal graph representation method incorporating embedded signal features and distance relationships, achieving a remarkable Macro F1 score of 88.61% in classifying normal, supraventricular ectopic, and ventricular ectopic beats.
▪ We validate our approach using real-world data from 328 patients with a commercial patch-type single-lead ECG device, demonstrating its scalability and robustness in clinical settings.

## Introduction

Electrocardiogram (ECG) analysis is a primary method for diagnosing and monitoring heart conditions.^1^^,^^2^ In recent years, the application of deep learning to ECG analysis has seen remarkable success, significantly enhancing diagnostic performance and efficiency.^3^, ^4^, ^5^, ^6^, ^7^, ^8^ Notably, these deep learning approaches have reached a point where they can outperform expert cardiologists in diagnostic accuracy for specific conditions.^9^^,^^10^ Among the various deep learning techniques, models such as multilayer perceptrons (MLPs), convolutional neural networks (CNNs), recurrent neural networks (RNNs), long short-term memory networks (LSTMs), and Transformers have made substantial contributions in detecting and classifying arrhythmias, including atrial fibrillation and ectopic beats.^7^^,^^11^^,^^12^ Detailed descriptions of these neural network approaches are provided in Section S1 of Supplementary Information (SI).

Graph convolutional networks (GCNs)^13^ present a promising alternative. Unlike CNNs, RNNs, and LSTMs, which require fixed-length inputs, GCNs are inherently flexible in handling sequences of varying lengths, making them especially well-suited for processing real-world ECG signals of diverse durations.^14^^,^^15^ GCNs excel in extracting features from nodes and edges, allowing them to model complex relationships in the data. They are gaining increasing recognition in the ECG field as an effective method for training on the morphological and rhythmic information of ECG signals.^14^^,^^16^, ^17^, ^18^, ^19^ By representing ECG signals as graphs, where nodes correspond to individual signal segments (eg, P wave, QRS complex, and T wave) and edges capture their interrelationships, GCNs can efficiently learn both spatial and temporal patterns.^14^^,^^15^ This unique capability enables GCNs to more accurately detect arrhythmias by effectively capturing intricate dependencies between heartbeats. Key features essential for arrhythmia diagnosis, including P wave, QRS complex, and T wave (collectively termed P-QRS-T) analysis and interval relationships, are detailed in Section S2 of SI. Previous studies have explored GCNs in ECG analysis. For instance, Duong et al.^20^ transformed ECG waveform images into graphs for feature extraction and model training. Ma and Xia^14^ proposed a batch processing technique to predict the presence of atrial fibrillation using graph representations of ECG signals.

Conventional graph pooling methods, such as add pooling, max pooling, and average pooling,^14^^,^^21^^,^^22^ face limitations when processing graphs with varying numbers of nodes (ie, different numbers of beats per graph), often resulting in the loss of crucial beat-specific information. To address these limitations, we present a QRS-centered weighted average pooling method that restructures data around the QRS complex, enabling effective beat-to-bzeat classification in a many-to-many format. Building on this, we devise a graph representation in which each P-QRS-T segment is treated as an individual node, and class imbalance is mitigated via graph data augmentation. Unlike previous approaches constrained by fixed-length inputs, our GCN model flexibly processes variable-length ECG signals. We evaluate whether this flexibility improves performance on real-world clinical data, where signal lengths vary substantially. Finally, this study introduces the Electrocardiogram Graph Convolutional Network (ECG-GraphNet), which leverages GCNs to detect and classify ectopic beats.

Our method is a new approach that uses P-QRS-T information to represent ECG signals as graphs and applies them to ECG-GraphNet. In this study, we conducted comprehensive experiments using the collected single-lead ECG data to achieve the following objectives:

1. Evaluate various ECG graph representation and augmentation methods to determine the most effective strategy for beat classification.
2. Compare the performance of different GCN architectures to design an optimal beat classification model.
3. Validate the scalability and clinical applicability of ECG-GraphNet through rigorous experiments.

The experiments conducted in this study illustrate the significant potential of ECG-GraphNet, which leverages graph-based deep learning techniques for arrhythmia diagnosis and ECG signal analysis. By demonstrating its effectiveness, our findings offer a novel perspective on utilizing graph structures in the medical field, particularly for analyzing complex sequential data such as ECG signals.

## Methods

The methodology of this study is structured around 3 central axes: (1) We propose and optimize key graph representation components—including node features, edge definitions, and a data augmentation method. (2) Based on the optimized components, we compare several candidate architectures to determine the final model configuration. (3) We rigorously validate the model to demonstrate its scalability and practical applicability. Through these procedures, we validate the research objectives outlined previously.

### Dataset

The research reported in this paper adhered to the Declaration of Helsinki guidelines for human research. This study was exempt from review by the Public Institutional Review Board of the Korea National Institute for Bioethics Policy, under the Ministry of Health and Welfare (P01-202408-01-013). Due to the Institutional Review Board exemption, the requirement for patient consent was waived. All patient data has been collected and processed in accordance with relevant data protection and privacy regulations.

ECG-GraphNet requires precise annotations of the onset and offset of each segment in the ECG data. The MIT-BIH database,^23^ which has been widely used in previous studies,^24^, ^25^, ^26^ does not provide precise segment location information. Moreover, it is unsuitable due to the excessively imbalanced distribution of heartbeat classes.^27^, ^28^, ^29^ In this study, to overcome the limitations of existing datasets such as MIT-BIH, we constructed a new dataset by collecting single-lead ECG data from 328 patients using a patch-type single-lead ECG recording device. The precise onset and offset of each P-QRS-T segment were annotated by clinical technicians, who labeled each heartbeat. Clinical technicians were trained to ensure the precision of annotations, and multiple rounds of validation were conducted to maintain data quality.

Each patient’s ECG signals were segmented into 10-second intervals and initially reviewed by clinical technicians, followed by a secondary review by a cardiologist. In this study, each 10-second ECG segment was classified into 3 heartbeat types: normal beats (*N*), supraventricular ectopic beats (S), and ventricular ectopic beats (V). Additionally, segments exhibiting intraventricular conduction delays, right bundle-branch block, or left bundle-branch bloc were excluded from the study. As a result, the final dataset consisted of 1253 10-second ECG segments collected from 328 patients, amounting to a total of 17,526 beats. Detailed data preprocessing is provided in Section S3 of SI.

### Optimization of ECG graph representation

**Figure 1:**
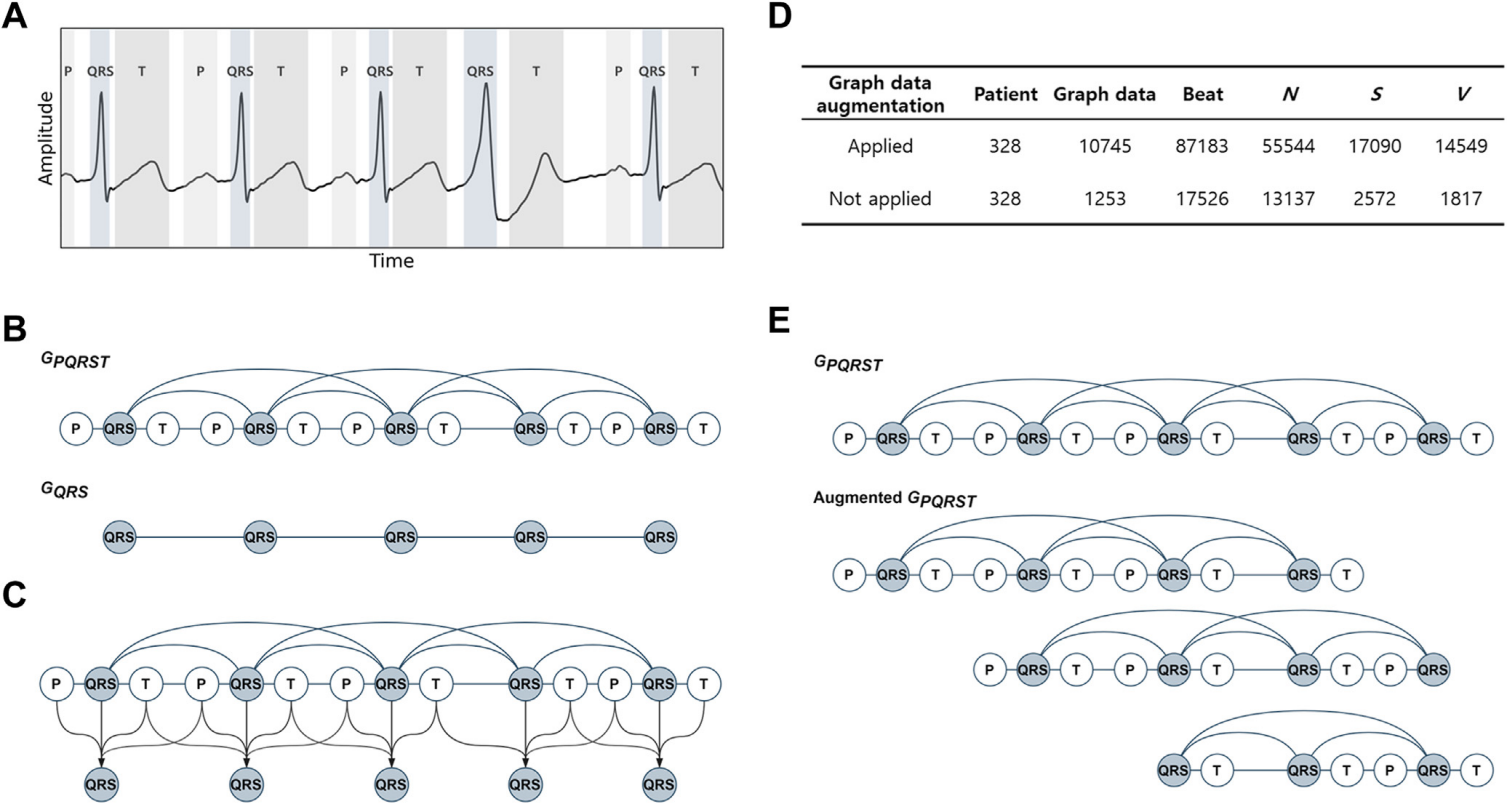
The ECG graph representation methods. **A:** ECG data showing the location of P-QRS-T. **B:** Schematic illustration of *G*_*PQRST*_ and *G*_*QRS*_. **C:** Schematic illustration of QRS-centered weighted average pooling method. **D:** Schematic illustration of the graph data augmentation method. **E:** Changes in data distribution by applying the graph data augmentation method.

The primary objective of ECG signal analysis is to accurately interpret the electrical activity of the heart.^30^ Each heartbeat consists of 3 primary segments—P-QRS-T—that represent distinct cardiac events. As shown in Figure 1A, analyzing these segments together is essential for accurate diagnosis.^31^^,^^32^ To facilitate such analysis, this study introduces a graph-based representation of ECG signals, where each P-QRS-T segment is represented as an independent node. This approach, illustrated in Figure 1B, transforms sequential ECG segments into graph structures that represent both temporal and structural relationships.

#### Node feature matrix

The node feature matrix *X* is designed to encapsulate key characteristics necessary for an effective graph representation of ECG signals. The basic structure of *X* includes one-hot encoded vectors to differentiate among P-QRS-T segments, as well as the segment widths. To enhance the representation, we incrementally integrated 2 additional features—signal embeddings and distance features—as evidenced by the ablation study detailed in the Results section and summarized in Table 1.

**Table 1:** Comparison results of ECG graph representation methods

Method			5-fold cross-validation performance (%)										
			Total		N			S			V		
Edge	Signal embedding	Distance feature	Acc	Macro F1	Sn	PP	F1	Sn	PP	F1	Sn	PP	F1
QRS-QRS	Applied	Applied	91.83 (±2.13)	85.00 (±2.48)	93.75 (±2.09)	98.47 (±0.70)	96.05 (±1.33)	84.34 (±5.77)	77.98 (±4.40)	81.00 (±4.52)	86.62 (±6.81)	70.86 (±6.27)	77.94 (±6.55)
		Not applied	89.82 (±2.59)	82.39 (±4.36)	92.92 (±2.56)	96.35 (±1.50)	94.59 (±1.29)	75.06 (±10.66)	72.50 (±6.21)	73.59 (±8.16)	87.38 (±6.23)	72.53 (±10.34)	78.98 (±6.64)
	Not applied		89.16 (±3.37)	79.47 (±4.50)	93.16 (±3.74)	97.91 (±0.78)	95.45 (±2.08)	73.15 (±7.88)	70.21 (±13.45)	71.40 (±9.82)	81.83 (±7.63)	64.02 (±11.57)	71.57 (±8.02)
P-P			85.19 (±4.91)	73.28 (±6.29)	90.50 (±4.63)	96.31 (±0.80)	93.29 (±2.55)	59.16 (±12.78)	56.99 (±10.91)	57.95 (±11.86)	81.17 (±12.46)	60.04 (±11.30)	68.59 (±10.73)
QRS-QRS & P-P			87.05 (±3.86)	76.73 (±6.56)	91.65 (±3.71)	96.21 (±0.87)	93.86 (±2.24)	66.07 (±8.53)	64.76 (±12.12)	65.23 (±9.71)	81.43 (±12.46)	63.30 (±11.08)	71.10 (±11.75)

**Figure 2:**
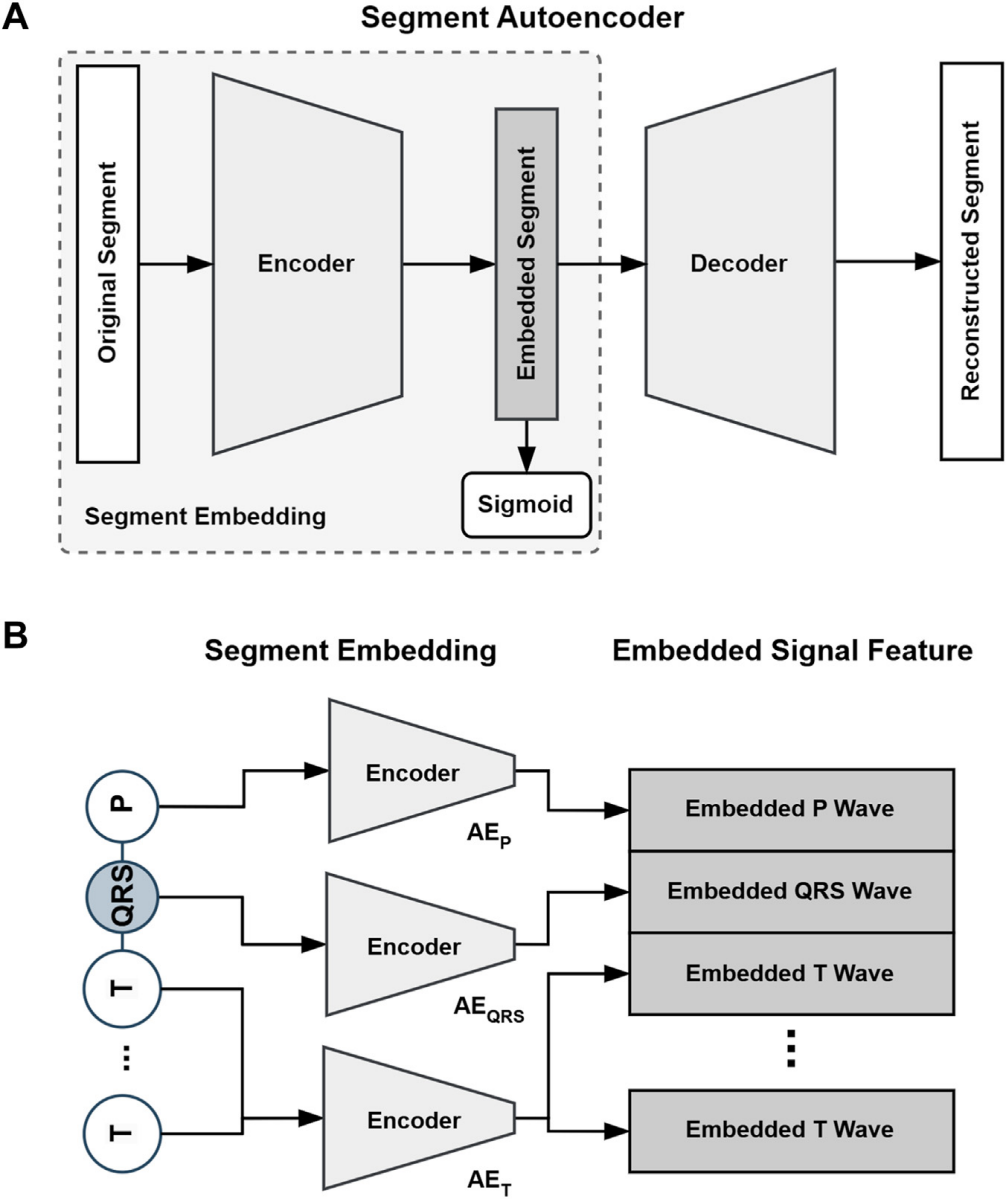
The signal waveform embedding method. **A:** Schematic diagram of AE for signal waveform embedding. **B:** The process of generating embedded signal features from segments using each AE.

Signal embeddings are low-dimensional latent vectors derived from the waveform of each segment. Autoencoders (AEs) were employed to derive these embeddings, as illustrated in Figure 2. AEs are deep learning models that compress high-dimensional data into a latent space and subsequently reconstruct the original input, preserving its principal characteristics.^32^ Individual AEs were designed for each segment type (AE_P_, AE_QRS_, AE_T_) to capture the unique waveform features of the P, QRS, and T segments, respectively. The latent vectors, scaled to a range between 0 and 1 using a sigmoid function, were then incorporated into the node feature matrix, as depicted in Figure 2B. Further details about the AEs are provided in Section S4 of SI.

Distance features capture temporal relationships by quantifying the time interval between the end of 1 segment and the beginning of the next. These temporal features were included alongside the AE-derived morphological features to represent both the timing and shape characteristics of ECG signals.

#### ECG graph construction

The ECG graph $G_{pqrst}=\left(X,A_{e}\right)$ represents the structural and temporal characteristics of ECG signals as a graph. In this representation, *X* denotes the node feature matrix, which contains the features extracted for each P, QRS, and T segment, while *A*_*e*_ represents the adjacency matrix that encodes the relationships between these nodes.

As illustrated in Supplemental Figure 1, for an ECG graph consisting of P-QRS-T segments, *X* has dimensions M × d, where *M* is the total number of nodes and *d* is the number of features per node. In this study, *d* was expanded to 22 through the inclusion of additional features as described previously. The symmetric matrix *A*_*e*_ defines the connectivity between nodes and assigns edge weights via a sigmoid-based function detailed in Section S5 of SI. This weighting mechanism ensures that segments closer in time are given higher weights, reflecting their stronger temporal correlation.

#### QRS-centered weighted average pooling

The variability in the number of beats within ECG data of the same length introduces significant challenges in processing,^33^^,^^34^ particularly when using GCNs. Traditional methods often address this issue by pooling the entire information of the ECG graph into a single node through techniques such as add pooling, max pooling, and average pooling.^21^^,^^22^ Advanced approaches, including differentiable pooling,^35^ top-k pooling,^36^, ^37^, ^38^ and self-attention graph pooling,^39^ pool data into unspecified nodes. However, these methods struggle to analyze ECG data on a beat-by-beat basis, which is crucial for arrhythmia detection.

To overcome these limitations, we propose a QRS-centered pooling method. This approach aggregates node features at the beat level by emphasizing the QRS complex. As the QRS complex plays a critical role in arrhythmia detection, this method restructures the graph data around it. QRS-centered pooling computes a weighted average of features from the central QRS node and its surrounding nodes, and it is defined as:

$$QRS-CenteredPool\left(f_{n}\right)=\frac{1}{1+k+j}\sum_{i=-k}^{j}\left(1-c\times |i|\right)f_{n+i}$$

Here, *f*_*n*_ represents the feature vector of the *n-*th QRS node, and *c* is a constant weight factor. The indices *k* and *j* denote the positions of the nearest preceding and following QRS nodes in chronological order relative to the *n-*th node. As shown in Figure 1C, nodes further from the central QRS node receive progressively smaller weights. The resulting weighted features are then assigned to the central node, thereby enhancing its representation with contextual information from adjacent P and T nodes.

#### Graph data augmentation

As shown in Figure 1D, the dataset used in this study was significantly imbalanced in terms of heartbeat class distribution. Class *N* accounted for 75.0% of the total data, while classes *S* and *V* constituted only 15.2% and 10.4%, respectively. This imbalance is a well-recognized challenge in ECG data analysis, often leading to performance degradation and overfitting in deep learning models.^40^ To address this issue, we developed and applied a graph data augmentation method specifically designed to enhance the representation of the minority classes, *S* and *V*, as shown in Figure 1E. A comprehensive algorithmic description of the graph data augmentation method is provided in Section S6 of SI, and its effect on model performance is thoroughly analyzed in the Results.

### Optimization of ECG-GraphNet architecture

#### Graph convolutional networks

The *G*_*PQRST*_ includes nodes representing the P-QRS-T segments, with an adjacency matrix that reflects both structural and temporal relationships between these nodes, as described previously. This graph enables the model to learn inter-segment and inter-beat relationships, which are critical for comprehensive ECG signal analysis.

Among the P-QRS-T segments, the QRS complex is particularly significant for analyzing ECG signals,^41^, ^42^, ^43^, ^44^, ^45^ and RR interval (time between 2 successive R peaks, termed RRI) is a key characteristic widely used for heartbeat variation detection in ECG research.^29^^,^^41^^,^^46^, ^47^, ^48^ Based on this foundation, this study introduces *G*_*QRS*_, a specialized QRS-only graph, as shown in Figure 1B. In *G*_*QRS*_, each node corresponds to a QRS complex, and edges are established only between consecutive QRS nodes. The edge weights are derived from the RRI, capturing temporal changes between successive beats.

The graph convolution (GC) layer processes *G*_*PQRST*_ and *G*_*QRS*_ as described in Section S7. The fully connected (FC) layer applies a linear transformation to the pooled graph vectors. Batch normalization (BN) follows each layer to stabilize training and mitigate overfitting, while a rectified linear unit (ReLU) activation function introduces non-linearity and enhances the model’s representational capacity. A detailed description of the model architecture can be found in Section S7 of SI.

#### ECG-GraphNet architectures

**Figure 3:**
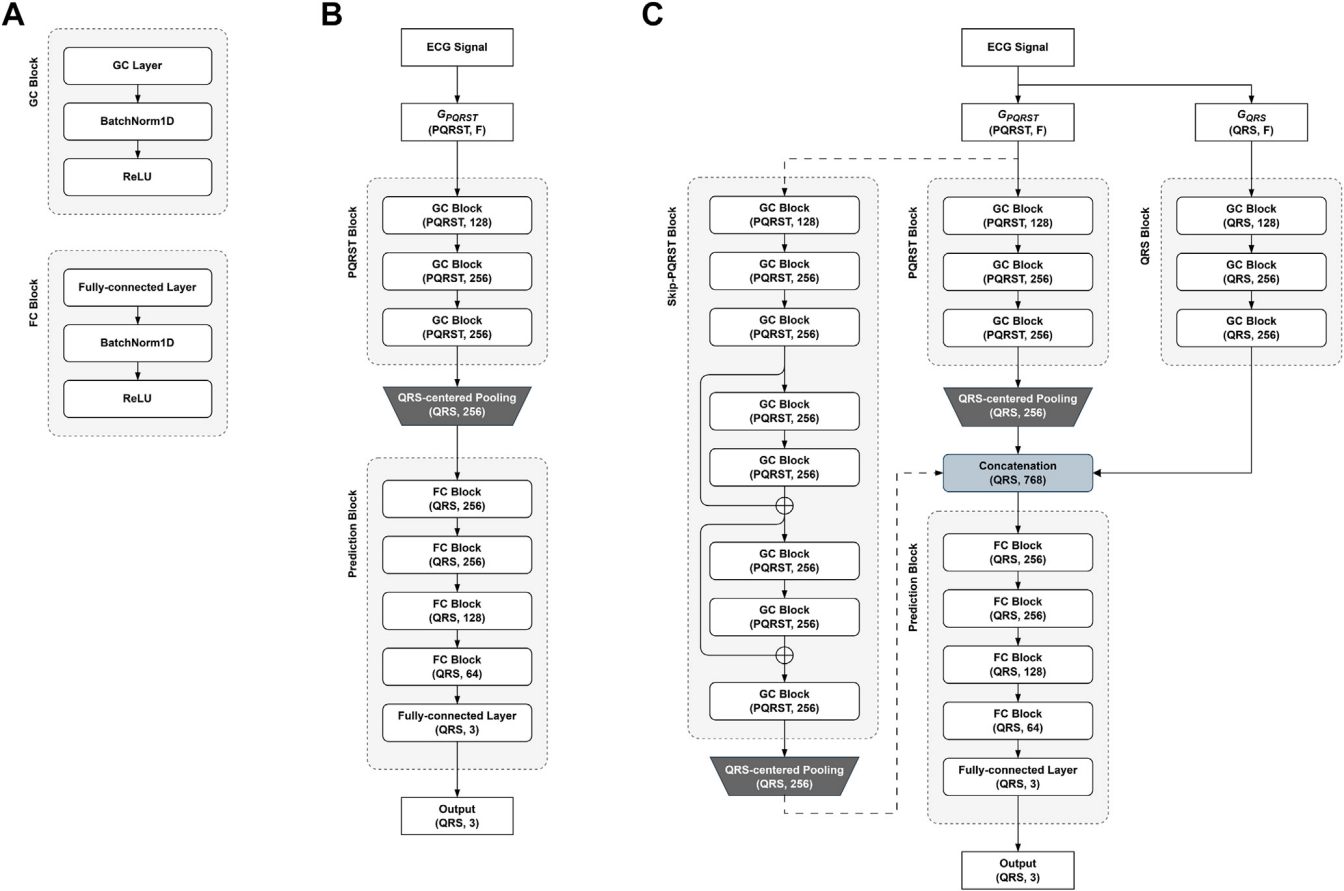
Three GCN architectures used in the experiments. **A:** Structures of the GC block and FC block that constitute the basic architecture. **B:** Architecture of PQRST-Net. **C:** Architecture of PQRST-QRS-Net if it does not include Skip-PQRST block (connected by a *dotted line*), and architecture of ECG-GraphNet if it does include Skip-PQRST block. Here, parentheses refer to (input channel, output channel), *F* refers to the feature dimension of the node feature matrix, PQRST refers to the number of P-QRS-T, and QRS refers to the number of QRS complexes.

As shown in Figure 3A, ECG-GraphNet is designed as a block consisting of several core layers. Three distinct GCN architectures were designed to identify the most effective architecture, as illustrated in Figure 3. The 3 architectures are:

• PQRST-Net: PQRST block & QRS-centered pooling & Prediction block
• PQRST-QRS-Net: PQRST-Net & QRS block
• ECG-GraphNet: PQRST-QRS-Net & Skip-PQRST block

The comparative performance results of these architectures are provided in the results.

### Evaluation

All experiments in the optimization process were conducted using the 5-fold grid search cross-validation (CV) with an interpatient split,^49^ ensuring no overlap of ECG data from the same patient between the training and validation sets. The grid search method for ECG-GraphNet is described in Table S1. Model performance was evaluated using the following metrics: sensitivity (Sn), positive predictive value (PPV), accuracy (Acc), F1 score, and Macro F1 score. A detailed explanation of these metrics is provided in Section S9 of SI. Accordingly, the best results were determined based on the average Macro F1 score across the 5-fold CV, where the error range was defined as (maximum score – minimum score)/2. To ensure robustness, all models employed the cross-entropy loss function, Adam optimizer, and early stopping based on the validation set loss.

### Scalability verification approach

In addition to identifying the optimal architecture, this study evaluated the scalability and potential of ECG-GraphNet through the following hypotheses: Hypothesis (1) ECG-GraphNet, when trained on various ECG patterns, will correctly classify new, unseen ECG patterns; Hypothesis (2) ECG-GraphNet will generalize to ECG graphs of larger sizes than those used during training.

Unlike the optimization experiments described previously in Methods, we newly split the data into distinct training and test groups to rigorously verify these hypotheses, while still maintaining an interpatient split to prevent data leakage. Patients who possessed at least one 8-beat graph containing ≥ 1 *N* beat and ≥ 4 consecutive *S* or *V* beats were designated as test patients; all remaining patients were designated as training patients. From these patient groups, we constructed 1 training set and 3 test sets: the 8-beat training set, the Pattern test set, the Size test set, and the Control test set, as detailed in Section S8 of the SI.

The datasets are summarized as follows:

• 8-beat training set: contains only 8-beat graphs and their augmented graphs from training patients.
• Pattern test set: contains graphs from test patients that include ≥ 1 *N* beat and ≥ 4 consecutive *S* or *V* beats, forming patterns (eg, *NNNNSSSS*, *NNNVVVVN*) that are not observed in the 8-beat training set.
• Size test set: contains 10-second graphs from test patients with ≥ 9 beats, that is, graphs larger than those seen in the 8-beat training set.
• Control test set: contains graphs similar in pattern and size to those in the 8-beat training set, but derived from test patients.

Model training was conducted using 5-fold CV applied only within the 8-beat training set to ensure robust model selection, resulting in 5 independently trained models. For the evaluation of the 3 test sets (Pattern, Size, and Control), the predictions from the 5 models were combined using a soft-voting ensemble strategy. Specifically, the softmax probability outputs of all models were averaged, and the final class prediction was determined by selecting the class with the highest average probability. A detailed analysis of these scalability evaluation results is provided in the Results.

## Results

### Optimization of ECG graph representation

#### Comparison of graph representation methods

A series of experiments were conducted in this study to determine the optimal structure of *G*_*PQRST*_ for representing ECG signals. The experiments focused on enhancing edge configurations, incorporating waveform features, and adding distance relationships to the node feature matrix. This section evaluates the performance impact of different graph representation methods introduced in the Methods.

The first experiment examined the effect of introducing edges between nodes of the same type within *G*_*PQRST*_. Initially, *G*_*PQRST*_ was defined with edges only connecting sequentially adjacent nodes. Additional configurations were then tested by incorporating edges between nodes of the same type, such as P-P and QRS-QRS edges, within a predefined window size. As shown in Figure 1B, when the window size was set to 5, the third QRS node was symmetrically connected to the 2 QRS nodes on either side, forming a total of 5 nodes, including itself.

Three edge configurations were compared: (i) using only QRS-QRS edges, (ii) using only P-P edges, and (iii) using both QRS-QRS and P-P edges with a window size of 5. The performance comparisons revealed that the graph with both QRS-QRS and P-P edges improved the Macro F1 score from 73.28% (±6.29) to 76.73% (±6.56) compared to the graph with only P-P edges. The graph with only QRS-QRS edges achieved the highest score of 79.47% (±4.50). Based on these results, the configuration for *G*_*PQRST*_ included only QRS-QRS edges within the maximum window size.

The second experiment evaluated the impact of representing the waveforms of each segment type as node features of *G*_*PQRST*_ using AEs. The GCN model with node features consisting solely of one-hot encoded identifiers and widths was compared to the GCN model that additionally included embedded signal features generated by AEs. The results showed that incorporating these embedded signal features improved the Macro F1 score to 82.39% (±4.36), an increase of approximately 2.92 percentage points (pp). Following this result, the node feature matrix was updated to include the embedded signal features of the waveforms.

The third experiment investigated the inclusion of distance relationships between sequentially adjacent nodes as additional features in the node feature matrix of *G*_*PQRST*_. Distance features represented the temporal distances between each node and its neighboring nodes. Comparing models with and without distance features showed that adding these features increased the Macro F1 score to 85.00% (±2.48), an improvement of 2.61pp. The F1 score for class *S* significantly improved from 73.59% (±8.16) to 81.00% (±4.52), a gain of 7.41pp. Based on these results, the node feature matrix was updated to include distance features. Table 1 summarizes the results of these experiments.

#### Effect of graph data augmentation

To address class imbalance, the data augmentation method described previously is applied and evaluated in this section. As shown in Figure 1D and detailed in Table S2, this study evaluated how applying the graph data augmentation method to the training set affected both data distribution and model performance. The primary objective was to address the class imbalance in the ECG dataset, thereby improving overall model performance. According to the results in Table 2, applying the graph data augmentation method led to improvements in model performance. Macro F1 score increased from 85.00% (±2.48) to 86.59% (±1.65). Furthermore, the F1 score for class *V* showed a significant gain of 3.99pp, improving from 77.94% (±6.55) to 81.93% (±4.77).

**Table 2:** Comparison results of the graph data augmentation method

Method	5-fold cross-validation performance (%)										
	Total		*N*			*S*			*V*		
Graph data augmentation	Acc	Macro F1	Sn	PP	F1	Sn	PP	F1	Sn	PP	F1
Applied	92.69 (±1.69)	86.59 (±1.65)	95.50 (±2.53)	97.27 (±1.19)	96.36 (±1.10)	84.73 (±2.57)	78.96 (±10.44)	81.47 (±5.02)	82.82 (±6.33)	81.48 (±8.16)	81.93 (±4.77)
Not applied	91.83 (±2.13)	85.00 (±2.48)	93.75 (±2.09)	98.47 (±0.70)	96.05 (±1.33)	84.34 (±5.77)	77.98 (±4.40)	81.00 (±4.52)	86.62 (±6.81)	70.86 (±6.27)	77.94 (±6.55)

### Comparison of GCN architectures

Based on the optimized graph representation components described above, we compared 3 GCN architectures to identify the most effective model design: PQRST-Net, PQRST-QRS-Net, and ECG-GraphNet, described previously and illustrated in Figure 3. PQRST-Net comprises PQRST block, PQRST-QRS-Net additionally includes QRS block, and the final architecture, ECG-GraphNet, also incorporates Skip-PQRST block. Consequently, as shown in Table 3, ECG-GraphNet achieved the highest performance among the 3 model architectures in a 5-fold CV with a Macro F1 score of 88.61% (±2.80). Additionally, due to potentially ambiguous segment boundaries, excluding the first and last beats of each segment during evaluation increased the Macro F1 score by 0.76pp from 88.61% (±2.80) to 89.37% (±2.92).

**Table 3:** Comparison results of GCN architectures

Method		5-fold cross-validation performance (%)										
		Total		*N*			*S*			*V*		
Architecture	Excluding both ends∗	Acc	Macro F1	Sn	PP	F1	Sn	PP	F1	Sn	PP	F1
ECG-GraphNet	Applied	93.87 (±1.88)	89.37 (±2.92)	95.67 (±1.92)	98.08 (±0.92)	96.86 (±0.92)	89.09 (±2.36)	83.62 (±6.09)	86.20 (±4.33)	87.65 (±8.32)	82.74 (±4.21)	85.06 (±5.10)
	Not applied	93.52 (±1.83)	88.61 (±2.80)	95.21 (±1.44)	98.09 (±0.78)	96.62 (±0.89)	88.14 (±2.14)	80.64 (±6.95)	84.16 (±4.68)	88.17 (±7.01)	82.23 (±3.61)	85.05 (±4.45)
PQRST-QRS Model		93.15 (±2.16)	87.87 (±3.60)	94.60 (±2.19)	98.43 (±0.22)	96.47 (±1.19)	88.24 (±2.90)	80.02 (±8.43)	83.81 (±4.79)	89.37 (±3.87)	78.38 (±11.14)	83.32 (±7.64)
PQRST Model		92.69 (±1.69)	86.59 (±1.65)	95.50 (±2.53)	97.27 (±1.19)	96.36 (±1.10)	84.73 (±2.57)	78.96 (±10.44)	81.47 (±5.02)	82.82 (±6.33)	81.48 (±8.16)	81.93 (±4.77)

∗ Excluding both ends means that both end beats are excluded from the performance evaluation.

### ECG-GraphNet scalability verification

To verify the generalization and scalability of ECG-GraphNet, we evaluated this model under rigorous test settings as described previously. The size of *G*_*PQRST*_ is determined by the number of segments and beats in the graph data, rather than by the timing of the recorded ECG signals. This property necessitates confirming that *G*_*PQRST*_ can accommodate variations in node and edge configurations as well as overall graph sizes across datasets. To evaluate the scalability of ECG-GraphNet, this study investigated its performance on ECG graphs of varying patterns and sizes, verifying its universal applicability.

According to Table 4, the Macro F1 score for the 5-fold CV of 8-beat training set was 86.54% (±2.39), closely matching Control test set at 88.40%. Similarly, Pattern test set and Size test set showed comparable scores of 85.21% and 87.03%, respectively. For a rigorous comparison, exact match accuracy, which measures the alignment of all 8-beats in a graph, was also calculated. The performance for 8-beat training set and Control test set was 64.53% (±6.67) and 66.71%, respectively, while Pattern and Size test sets achieved slightly lower scores at 48.60% and 49.83%, respectively.

**Table 4:** Scalability verification results for ECG-GraphNet

Dataset	Total	N	S	V	Exact match accuracy∗ (%)
	Macro F1 (%)	F1 (%)			
8-beat training set	86.54 (±2.39)	96.98 (±1.16)	77.27 (±6.57)	85.37 (±3.16)	64.53 (±6.67)
Pattern test set	85.21	87.98	84.47	83.19	48.60
Size test set	87.03	95.70	82.89	82.52	49.83
Control test set	88.40	96.74	85.23	83.21	66.71

∗ Exact match accuracy is the accuracy with which all beats in the graph are completely matched.

The performance evaluation was also conducted by excluding 1 beat at each end, as described in Table S3. The exact match accuracy with both ends excluded significantly improved across all datasets, achieving 73.95% (±5.58), 57.84%, 56.87%, and 76.97% for the 8-beat training set, Pattern test set, Size test set, and Control test set, respectively. Supplemental Figure 2 further illustrates the overall improvement in the performance of ECG-GraphNet across all beat patterns in Pattern test set, with the exact match accuracy increasing by 9.23pp compared to the accuracy reported in Table 4.

## Discussion

This study proposes and validates ECG-GraphNet, a novel methodology based on GCN for ECG beat classification. The discussion is structured around 3 central axes: (1) development and optimization of ECG graph representation methods, (2) evaluation and selection of effective model architectures, and (3) evaluation of model scalability and potential through rigorous verification. This study interprets the research results by considering these 3 axes and discusses their implications for practical applicability.

### Optimization of ECG graph representation methods

The optimal configuration of *G*_*PQRST*_ as proposed in this study comprises P-QRS-T nodes with self-loops, adjacent edges, and QRS-QRS edges. The node feature matrix incorporates identifiers, widths, embedded signal features, and distance features. Each edge is assigned a larger weight when the connected nodes are closer together. This ECG graph representation method effectively captures the structural presence and temporal relationships of each segment and beat, offering a robust foundation for ECG analysis. These findings suggest that this method has significant potential for extension, as it allows for the incorporation of additional segment relationships, such as node features, edge weights, and waveform embeddings.

Based on the optimized ECG graph representation components, we show that the proposed graph data augmentation method effectively improves model performance when applied to imbalanced ECG datasets. In particular, this approach addresses the class imbalance problem using graph-based representations without signal distortion.

### Comparative evaluation of model architectures

From the perspective of the model architecture, ECG-GraphNet, which integrates the PQRST block, QRS block, and Skip-PQRST block, achieved the best performance among the evaluated models. This result indicates that combining both segment-level and global signal-level representations within a unified architecture leads to superior classification performance.

Moreover, due to the temporal characteristics of the ECG signal, nodes located at both ends of the graph tend to exhibit low connectivity, which can negatively affect performance. To verify this effect, nodes at both ends were excluded from the evaluation, resulting in an increase in Macro F1 score by 0.76 pp. This suggests that it is important to consider connectivity bias in the ECG graph representation.

### Generalization and scalability of ECG-GraphNet

In this experiment, we demonstrate that ECG-GraphNet can accurately classify previously unseen ECG patterns and effectively handle larger, more complex graphs than those encountered during training. These findings indicate that ECG-GraphNet is well-suited for application to longer ECG recordings, even if they contain previously unseen patterns, highlighting its robustness and adaptability in real-world clinical applications.

Clinically, consecutive occurrences of *S* or *V* in ECG data are classified as supraventricular tachycardia (SVT) or ventricular tachycardia (VT), with the frequency of these sequences varying significantly among individuals.^50^^,^^51^ Scalability verification experiments suggest that ECG-GraphNet can accurately identify transition points where *N* changes to *S* or *V* and effectively evaluate ECG signals containing beats that follow previously unobserved patterns. Additionally, F1 scores for classes *N*, *S*, and *V* were nearly equivalent between the 8-beat training set and Control test set, indicating that ECG-GraphNet maintained consistent performance even when applied to unseen data patterns and varying sizes.

Furthermore, ECG-GraphNet is unaffected by graph size, enabling its application to long data sequences regardless of the measured ECG signal length. This flexibility, combined with its efficiency in managing structural and computational challenges, makes ECG-GraphNet relatively superior to other deep learning models. These findings demonstrate the superiority of ECG-GraphNet in ECG analysis, and we anticipate that it will make a significant contribution to the advancement of deep learning-based ECG analysis methods.

### Clinical applicability and limitations

This study proposed a novel method for transforming ECG data into a graph format and developed ECG-GraphNet, a model that classifies each heartbeat as *N*, *S*, or *V*. ECG-GraphNet was validated using data collected from a commercial patch-type single-lead ECG measurement device, demonstrating its applicability in real clinical settings. However, a limitation of this model is the requirement to detect the onset and offset of each P-QRS-T segment before transforming ECG signals into a graph. Despite many prior studies^52^^,^^53^ that have attempted to address this challenge, it remains a significant hurdle.

To overcome this limitation, we propose 2 potential approaches for future work. First, integrate established segment detection methods with ECG-GraphNet to create an end-to-end system. This integration would automate the preprocessing step and enhance the model's practical utility. Second, incorporate uncertainty estimation in the segment boundary detection process to improve robustness. These enhancements are expected to substantially improve the clinical applicability of our approach and facilitate its integration into existing ECG monitoring systems.

### Advantages over existing deep learning methods

ECG-GraphNet offers notable advantages, by addressing several inherent limitations of existing deep learning approaches. For example, MLPs rely heavily on feature engineering based on domain expertise, thus requiring specialized knowledge and limiting their generalizability. CNNs and hybrid models combining CNNs with RNNs, LSTMs, and Transformers require fixed-length input signals, making it challenging to process ECG signals of varying durations. In contrast, ECG-GraphNet accommodates variable-length sequences by representing them as graphs of different sizes and processes them effectively. Moreover, its ability to directly model relationships between nodes and edges enables the capture of interdependencies among P-QRS-T segments, a critical aspect of ECG analysis. However, as demonstrated in our experiments, converting ECG signals into graph representations necessitates meticulous design of nodes and edges, along with comprehensive validation to optimize associated hyperparameters. As a result, Table 5^54^, ^55^, ^56^, ^57^, ^58^, ^59^, ^60^, ^61^, ^62^, ^63^ summarizes the advantages and limitations of these MLPs, CNNs, RNNs, LSTMs, Transformers, and ECG-GraphNet. ECG-GraphNet provides a practical foundation for scalable ECG analysis, with the ability to handle variable-length inputs and capture clinically meaningful structures.

**Table 5:** Detailed comparison of ECG-GraphNet and previous studies on its advantages and limitations in ECG analysis

Model	Advantages	Limitations	References
MLP	• It provides a simple structure that can be easily implemented on various hardware platforms. • It achieves fast training speeds when the temporal or spatial features of signals are preprocessed.	• It requires engineered features as input and relies on prior domain knowledge for feature extraction. • It is limited in analyzing long sequential data and is better suited for short-term, fixed feature-based analysis.	^54^^,^^55^
CNN	• It allows direct input of ECG signals to learn key patterns and features. • It can automatically extract useful features without the need for domain knowledge or extensive preprocessing.	• It has limitations when processing variable-length signals due to its fixed input length requirement. • It struggles to effectively capture temporal dependencies in sequential data.	^25^^,^^27^^,^^49^^,^^56^, ^57^, ^58^
RNN/LSTM	• It models temporal dependencies, making it advantageous for sequential data like ECG signals. • It reflects changes over time in signal patterns when given sequential data.	• It has constraints when processing variable-length signals due to its fixed input length requirement. • It requires a high computational cost, particularly with large-scale ECG datasets.	^7^^,^^11^^,^^25^^,^^26^^,^^57^^,^^59^
Transformer	• It facilitates parallel processing, which significantly reduces training and inference time for long input sequences. • It improves anomaly and arrhythmia detection by combining spatial and temporal feature processing when used with CNNs.	• It requires significant data and hardware resources due to its relatively complex model structure. • It requires fixed-length input signals when combined with CNNs.	^11^^,^^12^^,^^60^, ^61^, ^62^, ^63^
ECG-GraphNet	• Unlike models that require fixed-length input, it handles variable-length sequences. • It effectively captures interdependencies between segments by modeling relationships between nodes and edges.	• It is highly dependent on how nodes and edges are defined and constructed during the transformation of ECG data into graph form. • It requires extensive experiments and validation to identify optimal hyperparameters, such as node features and edge configuration.	^14^^,^^15^^,^^20^

CNNs = convolutional neural networks; LSTMs = long short-term memory networks; MLPs = multilayer perceptrons; RNNs = recurrent neural networks.

## Conclusions

In this study, we introduced ECG-GraphNet, a graph convolutional network designed to enhance arrhythmia classification by leveraging a novel graph representation of ECG signals. The key components of the ECG waveform, P-QRS-T, were represented as individual nodes, while the relationships between nodes were expressed as edges corresponding to each interval. Individual beats were classified using the QRS-centered pooling method, and performance was further enhanced through the data augmentation method. As a result, our model achieved a Macro F1 score of 88.61% on a challenging dataset of 10-second ECG recordings collected from 328 patients. Moreover, this approach demonstrated its adaptability to varying ECG patterns and signal lengths, effectively distinguishing *N*, *S*, and *V* beats.

Our findings indicate that ECG-GraphNet provides a promising solution for clinical diagnosis and long-term single-lead ECG monitoring of patients. The model’s ability to generalize across diverse ECG patterns and handle variable-length signals underscores its scalability to more complex datasets and clinical environments. ECG-GraphNet provides a robust foundation for advancing automated arrhythmia detection, with the potential to significantly enhance cardiovascular care and patient diagnostic outcomes.

Future research directions include: (1) developing an end-to-end framework that integrates segment detection and classification; (2) improving the graph structure to address connectivity bias; (3) extending the approach to multi-lead ECG analysis; and (4) improving model interpretability for clinical applications. These advances will enhance the utility of ECG-GraphNet for early diagnosis and monitoring of cardiac diseases in diverse patient populations.
