# Supplementary material for: ECG-GraphNet: Advanced arrhythmia classification based on graph convolutional networks
Source: Heart Rhythm O2. 2025 May 19;6(8):1199–211. doi: 10.1016/j.hroo.2025.05.012 (PMC12411959; doi:10.1016/j.hroo.2025.05.012)
Supplement: Appendix A — Supplementary Information [file mmc1.docx]

**SUPPLYMENTARY INFORMATION**

**ECG-GraphNet: Advanced Arrhythmia Classification Based on Graph Convolutional Networks**

Myeonghun Lee^1, +^, Jiwoo Lim^1, +^, JinKook Kim^1, *^

^1^HUINNO Co., Ltd., Seoul, Republic of Korea

+These authors equally contributed to this work.

^∗^Corresponding author: JinKook Kim (jinkook@huinno.com)

Brief title: ECG-GraphNet for Arrhythmia Classification

**S1 Neural networks for ECG processing**

Due to their simple architecture, MLPs are particularly suitable for deployment on various hardware systems and efficient in scenarios where preprocessed feature vectors are used for fast learning^1,2^. However, they require extensive feature engineering and rely heavily on domain expertise. On the other hand, CNNs have been proven effective in directly processing raw ECG signals, automatically extracting critical patterns and features, thereby reducing the need for manual feature extraction^3–7^. Despite their strengths, CNNs struggle with variable-length time-series data due to their requirement for fixed-length inputs and often fail to capture temporal dependencies in ECG signals^3–8^. RNNs and LSTMs, designed to model temporal dependencies, are effective in capturing time-varying patterns in ECG signals but are computationally intensive and inefficient when handling large ECG datasets due to their inability to parallelize computations and their requirement for fixed-length inputs^4,6,9–12^. Meanwhile, Transformers leverage parallel processing to manage long sequences efficiently and, when combined with CNNs, can capture both spatial and temporal features of ECG signals. However, Transformers require high computational costs due to their structural complexity, and when combined with CNNs, they also require fixed-length inputs^12–17^. As a result, this reliance on fixed-length inputs, as seen in previous studies, limits their applicability given that real ECG signals have highly variable lengths.

**S2 Key features for arrhythmia diagnosis**

Arrhythmia diagnosis requires detailed information about each segment of a single heartbeat, specifically the P wave, QRS complex, and T wave (collectively termed P-QRS-T)^18–20^. Each segment has specific roles: the P wave indicates atrial depolarization, the QRS complex reflects ventricular depolarization, and the T wave represents ventricular repolarization^20^. Additionally, ECG analysis must also account for beat-to-beat relationships such as the RR interval (the time between two successive R peaks, termed RRI) and segment-to-segment relationships like the PP interval, TP interval, and PR interval^21,22^. Furthermore, ECG analysis requires information about the presence and duration of each segment. Existing deep learning models often do not effectively incorporate these morphological, temporal, and relational features into their architecture, which can limit their diagnostic accuracy. Therefore, there is a growing need for new methodologies that can capture both inter-beat (beat-to-beat) and inter-segment (segment-to-segment) relationships to improve diagnostic performance^21,23,24^.

**S3 Details of data preprocessing**

In this study, the 10-second ECG data was classified into the following categories: normal sinus, atrial premature complexes (APC), ventricular premature complexes (VPC), atrial fibrillation (AF), supraventricular tachycardia (SVT), and ventricular tachycardia (VT). Notably, the AF category encompassed both atrial fibrillation and atrial flutter. 10-second ECG segments that did not fit these predefined categories were labeled as others.

After a comprehensive review, patients exhibiting arrhythmic events were randomly selected for inclusion in this study. However, AF data were excluded to avoid confounding effects due to highly irregular RRI, which can obscure the classification of ectopic beats^25^. Similarly, 10-second ECG segments exhibiting ventricular conduction delays, including IVCD, RBBB, and LBBB, were categorized as others and excluded from the study. This decision was made to prevent potential misclassification, as these conduction abnormalities can significantly alter QRS morphology, leading to normal beats (*N*) being misclassified as ventricular ectopic beats (V)^26,27^. The remaining data was automatically classified into three heartbeat types: *N*, supraventricular ectopic beats (*S*), and *V*. Beats that could not be classified were labeled as unclassified beats (*Q*), and any 10-second ECG segments containing *Q* beats were excluded. Following this process, the final dataset consisted of 1,253 10-second ECG segments collected from 328 patients, amounting to a total of 17,526 beats.

In this study, eight experts manually annotated the precise onset, offset, and R peaks for each P-QRS-T segment to enhance the accuracy of the analysis. The labeling process omitted P waves obscured by QRS complexes or T waves because their identification is challenging and susceptible to inter-observer variability. All experts underwent standardized training and adhered to strict guidelines to ensure consistency and accuracy in the annotations. This meticulous approach ensured high-quality annotations for subsequent analysis.

**S4 Autoencoders for signal embedding**

Autoencoders (AEs) were designed for embedding the P wave, QRS complex, and T wave, denoted as AE_P_, AE_QRS_, and AE_T_, respectively. Each AE consists of 4 encoder blocks and 4 decoder blocks, where each block comprises a fully connected (FC) layer, a batch normalization (BN) layer, and a leaky rectification linear unit (ReLU) activation function. Each segment had fewer than 128 dimensions, so the remaining part was zero-padded and converted into input vectors. Therefore, each AE reduced the dimensionality of segments from 128 dimensions to 16 dimensions. These AEs were trained using the mean absolute error (MAE) loss function, and the final trained models achieved MAE scores of 0.007, 0.015, and 0.018, for AE_P_, AE_QRS_, and AE_T_, respectively.

**S5 Edge weight calculation**

The adjacency matrix *A_e_* in the ECG graph represents the relationships between nodes by assigning weights to the edges. These weights are determined using a sigmoid-based function to represent the temporal distance between connected nodes. The edge weight *E(s)* is defined as:

$$E\left( s \right) = \frac{1}{1+e^{\left( ps-q \right)}}$$

Here, *s* represents the temporal distance between the offset of the preceding node and the onset of the following node, while *p* and *q* are constants that control the rate of weight decay. This function ensures that nodes closer in time are assigned higher weights, reflecting their stronger temporal relationships.

The *A_e_* is a symmetric *M* × *M* matrix, where *m* is the total number of nodes in the graph. It fundamentally includes self-loops for each node, ensuring that every node is connected to itself with a weight of 1. Bidirectional edges are established between consecutive nodes to represent the temporal sequence of the segments. The matrix structure is represented as:

$$A_{e}=\left( \begin{matrix} 1 & \cdots& E(a_{1m}) \\ \vdots& \ddots& \vdots\\ E(a_{m1}) & \cdots& 1 \end{matrix} \right)$$

In this matrix, *a_1m_* denotes the weight of the edge between the 1st and *m-*th nodes in a graph with *M* total nodes*.* By default, the graph structure includes self-loops and bidirectional edges. An additional experiment was conducted to optimize the adjacency matrix by introducing edges between nodes of the same type (e.g., P-P and QRS-QRS nodes) within a predefined temporal window size. The results of this experiment are detailed in **Section 3.1 Comparison of graph representation methods**. This approach allows the graph to incorporate both short- and long-range temporal dependencies, making it suitable for capturing the complex interactions present in ECG signals.

**S6 Details of graph data augmentation**

The proposed graph data augmentation method involves randomly removing nodes from the extremities of transformed ECG graphs, as shown in **Figure 1(d)**. By generating new graphs of different lengths and configurations, this technique creates a diverse set of graph samples that can be effectively processed by a GCN, allowing the model to learn diverse representations within the node feature matrix. To maintain the structural integrity of the ECG signals, each augmented *G_PQRST_* was carefully constructed to include at least three consecutive QRS nodes. The cuts were made exclusively between QRS nodes to preserve the coherence of the ECG signal. Additionally, graph data augmentation was performed only on the training set, as the patients in the training and validation sets were independently separated to avoid data leakage.

The graph data augmentation technique applied in this study can be mathematically expressed as follows:

| **Algorithm 1** Graph Data Augmentation Process for ECG Signals |
| --- |
| **Require:** *ECG_Records*, a collection of 10-second ECG records including *S* or *V*  **Ensure:** Augmented *ECG_Records* with a balanced distribution of *S* and *V*  1: **for** each *record* in *ECG_Records* **do**  2: **if** *record.beat label* ∈ {*S*, *V*} **then**  3: *selected_QRS* ← SELECTRANDOMQRS(*record*)  4: *num_QRS* ← GENERATERANDOMNUMBER  5: *augmented_record* ← CROPANDGENERATE(*record*, *selected_QRS*, *num_QRS*)  6: Append *augmented_record* to *ECG_Records*  7: **end if**  8: **end for**  9: **function** SELECTRANDOMQRS(*record*)  10: **return** a randomly selected QRS complex within *record* that matches *beat_label S* or *V*  11: **end function**  12: **function** GENERATERANDOMNUMBER  13: **return** a random integer representing the total number of QRS complexes to include in a new augmented graph  14: **end function**  15: **function** CROPANDGENERATE(*record*, *selected_QRS*, *num_QRS*)  16: Crop *record* by *num_QRS*, from their surroundings based on the *selected_QRS* to create a new graph  17: When cropping the *record*, cut between the QRS complexes and verify that the QRS complexes are complete  18: **return** a new augmented graph  19: **end function** |

This augmentation approach enhances training data diversity and mitigates class imbalance, allowing the model to more effectively learn the characteristics of underrepresented heartbeat classes

.

**S7 Details of graph convolutional networks**

The proposed ECG-GraphNet utilizes GCNs to model the complex structural and temporal patterns in ECG signals. The fundamental GCN operation^13^, as applied to *G_PQRST_* and *G_QRS,_* is defined as:

$$X^{(l+1)}=D_{e}^{-\frac{1}{2}}A_{e}D_{e}^{-\frac{1}{2}}X^{(l)}W^{(l)}$$

Here, *X^(l)^* represents the node feature matrix at the *l-*th layer, where each row corresponds to a node and encodes its features. *A_e_* is the weighted adjacency matrix, reflecting the connectivity and strength of relationships between nodes. *D_e_* is the weighted diagonal degree matrix, calculated by adjusting weights based on the number of edges connected to each node. *W^(l)^* denotes the trainable weight matrix at the *l-*th layer, used to transform node features. Finally, these components propagate and aggregate information between nodes, updating the feature matrix *X^(l)^* to *X^(l+1)^* at each layer. By iteratively updating features, the model captures both local and global dependencies, facilitating accurate arrhythmia detection through effective modeling of ECG signal patterns.

A GC block is composed of a GC layer, BN, and ReLU, while an FC block includes an FC layer, BN, and ReLU. Specifically, the GC block is further categorized into a PQRST block and a QRS block: the PQRST block is designed to model P-QRS-T relationships in *G_PQRST_*, while the QRS block focuses on modeling QRS relationships in *G_QRS_*. To enhance model depth and capacity, the larger graph *G_PQRST_* utilizes a Skip-PQRST block. This block introduces skip connections^28^, which allow features to bypass intermediate layers, enabling deeper graph convolutional operations and addressing the vanishing gradient problem. The final Prediction block comprises FC blocks that process the pooled graph vectors and generate the model’s output. This comprehensive architecture allows ECG-GraphNet to effectively extract and represent the essential features of ECG data, integrating both segment-level and beat-level information. Consequently, ECG-GraphNet provides a robust framework for precise ECG analysis, making it highly suitable for arrhythmia detection and classification.

**S8 Datasets for scalability verification**

To verify the scalability of ECG-GraphNet, the original 10-second data was newly separated and refined in the following steps and consisted of one training set and three types of test sets.

1. All data are divided into graphs of 8 beat units.
2. Among these, patients with at least one 8-beat graph consisting of at least one *N* and four or more consecutive *S* or *V* are defined as test patients.
3. Other than test patients, the rest are defined as training patients.
4. 8-beat training set
   1. The graph data augmentation method is applied to all 8-beat graphs of the training patients.
   2. 8-beat training set consists of all 8-beat graphs of training patients and their augmented graphs.
   3. The average performance of the 5-fold cross-validation (CV) of 8-beat training set is evaluated, and the augmented graphs are excluded from the validation sets.
   4. The five trained models are used to predict each of the test sets described below.
5. Pattern test set
   1. It consists of graphs from test patients that include at least one *N* beat and at least four consecutive *S* or *V*.
   2. These patterns are as follows:

*NNNNSSSS*, *NNNSSSSN*, *NNSSSSNN*, *NSSSSNNN*, *SSSSNNNN*, *NNNSSSSS*, *NNSSSSSN*, *NSSSSSNN*, *SSSSNNN*, *NNSSSSSS*, *NSSSSSSN*, *SSSSSSNN*, *NSSSSSSS*, *SSSSSSSN*, *NNNNVVVV*, *NNNVVVVN*, *NVVVVNN*, *NVVVVNNN*, *VVVVNNNN*, *NNNVVVVV*, *NNVVVVVN*, *NVVVVVNN*, *VVVVVNNN*, *NVVVVVV*, *NVVVVVVN*, *VVVVVVNN*, *NVVVVVVV*, *VVVVVVVN*

- 1. This dataset consists only of graphs with patterns that cannot be observed in 8-beat training set.
  2. Therefore, if the 5-fold CV performance of 8-beat training set and the prediction performance on the Pattern test set are similar, it supports *Hypothesis 1)* If ECG-GraphNet is trained on various ECG patterns, it can correctly classify new, unseen ECG patterns.

1. Size test set
   1. It consists of the original 10-second data graphs that include more than 9 beats from test patients.
   2. This dataset consists only of large-sized graphs that cannot be observed in 8-beat training set.
   3. Therefore, if the 5-fold CV performance of 8-beat training set and the prediction performance on the Size test set are similar, it supports *Hypothesis 2)* ECG-GraphNet can predict ECG graphs larger than those on which it was trained.
2. Control test set
   1. It consists of a graph of the remaining 8 beats that are not included in Pattern test set among the data from test patients.
   2. This dataset consists only of graphs from test patients that are similar to those in 8-beat training set.
   3. Therefore, if the 5-fold CV performance of 8-beat training set and the prediction performance on the Control test set are similar, this indicates that the experiments on the above Pattern test set and Size test set were properly derived.

**S9 Metrics**

In this study, given true positives (TP), false positives (FP), true negatives (TN), and false negatives (FN), the performance evaluation metrics for each beat class are defined as follows: sensitivity (Sn), positive predictive value (PP), and F1 score (F1).

$$\mathrm{Sn}=\frac{\mathrm{TP}}{TP+FN}$$

$$\mathrm{PP}=\frac{\mathrm{TP}}{TP+FP}$$

$$F1=\frac{2\times\mathrm{TP}}{2\times TP+FP+FN}$$

Additionally, when *n* represents the number of classes in the dataset, the performance evaluation metrics for the overall classification performance are as follows: accuracy (Acc) and Macro F1 score (Macro F1).

$$\mathrm{Acc}=\frac{TP+TN}{TP+TN+FP+FN}$$

$$\mathrm{Macro} F1=\frac{\sum_{i=1}^{n} {F1}_{i}}{n}$$

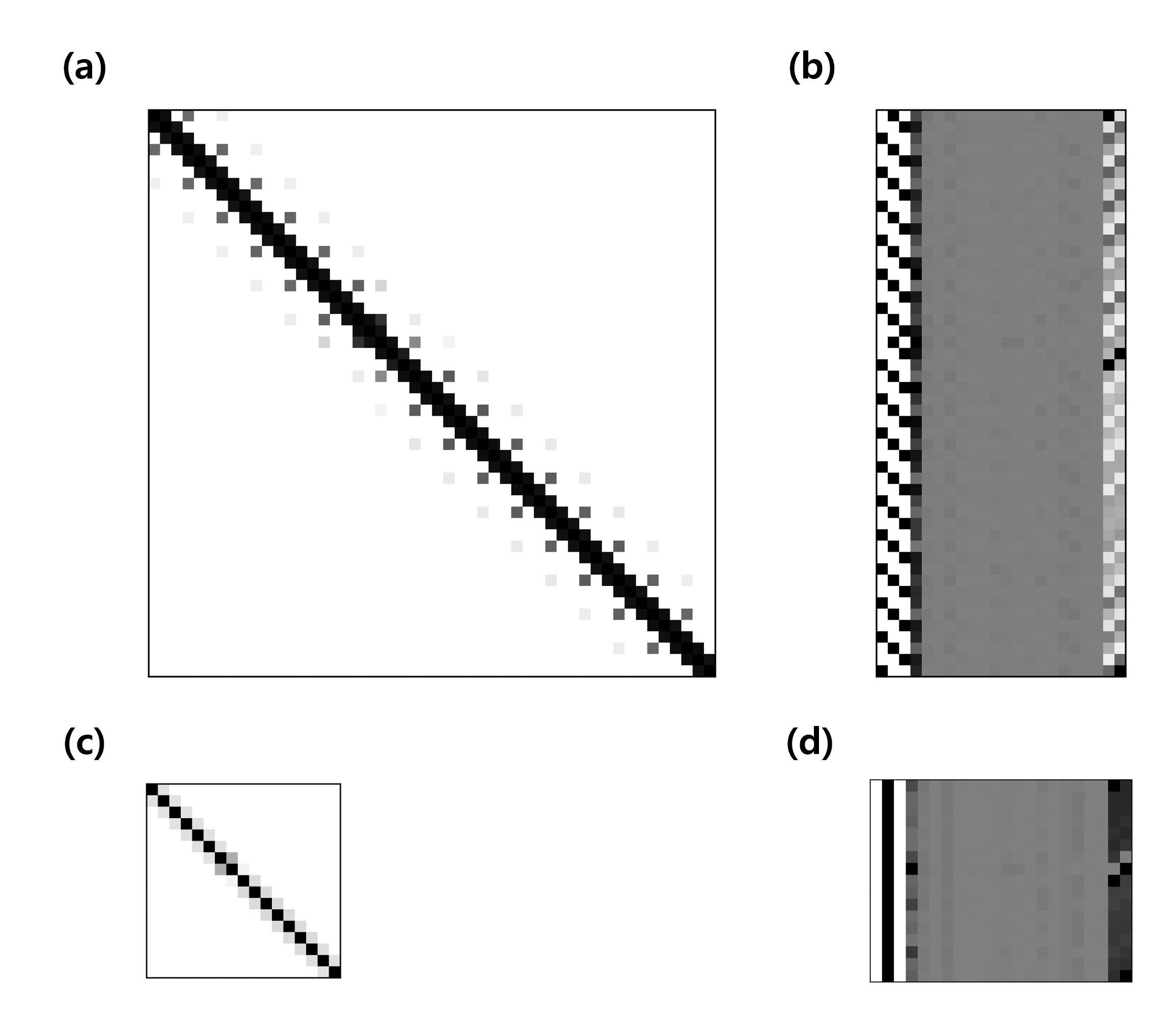


**Figure S1.** Visualization of node feature matrix and adjacency matrix of example ECG graph. **(a)** A node feature matrix of *G_PQRST_*. **(b)** An adjacency matrix of *G_PQRST_*. **(c)** A node feature matrix of *G_QRS_*. **(d)** An adjacency matrix of *G_QRS_*.

**
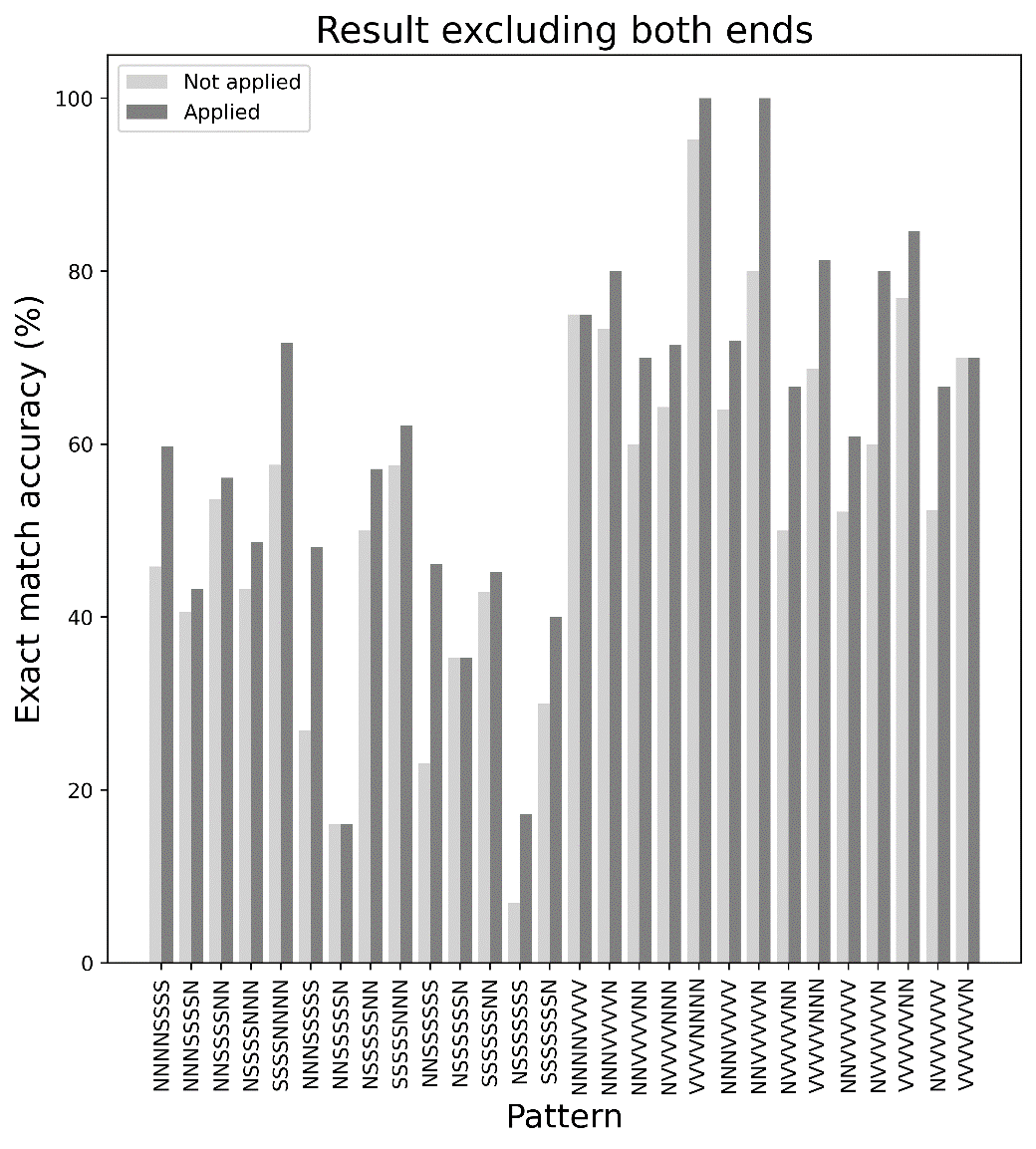
**

**Figure S2.** Exact match accuracy of ECG-GraphNet on Pattern test set. Performance by pattern (Not applied) and performance excluding both ends (Applied). Excluding both ends means that both end beats are excluded from the performance evaluation. Exact match accuracy is the accuracy with which all beats in the graph are completely matched.

**Table S1.** Hyperparameter Tuning Grid for ECG-GraphNet.

| **Hyperparameter** | **Grid** | **Best case** |
| --- | --- | --- |
| Learning rate | 0.001, 0.005 | 0.001 |
| (Number of PQRST blocks, Number of Skip-PQRST blocks) | (3, 10), (5, 10) | (3, 10) |
| Number of QRS blocks | 3, 5 | 5 |
| Dimensions of GC blocks | 128, 256, 512 | 256 |

**Table S2.** Distribution of the original and augmented datasets used in the 5-fold CV experiments.

| **Dataset** | **Graph data augmentation** | ***k*** | **Patient** | **Graph data** | **Beat** | ***N*** | ***S*** | ***V*** |
| --- | --- | --- | --- | --- | --- | --- | --- | --- |
| Training set | Applied | 1 | 262 | 8379 | 67338 | 42507 | 14294 | 10537 |
|  |  | 2 | 262 | 8701 | 71132 | 45342 | 14078 | 11712 |
|  |  | 3 | 262 | 8608 | 70743 | 45686 | 13836 | 11221 |
|  |  | 4 | 263 | 8538 | 69109 | 44092 | 12854 | 12163 |
|  |  | 5 | 263 | 8754 | 70410 | 44549 | 13298 | 12563 |
|  | Not applied | 1 | 262 | 982 | 13595 | 10141 | 2160 | 1294 |
|  |  | 2 | 262 | 1014 | 14241 | 10650 | 2125 | 1466 |
|  |  | 3 | 262 | 1017 | 14418 | 10893 | 2109 | 1416 |
|  |  | 4 | 263 | 996 | 13924 | 10455 | 1933 | 1536 |
|  |  | 5 | 263 | 1003 | 13926 | 10409 | 1961 | 1556 |
| Validation set | Not applied | 1 | 66 | 271 | 3931 | 2996 | 412 | 523 |
|  |  | 2 | 66 | 239 | 3285 | 2487 | 447 | 351 |
|  |  | 3 | 66 | 236 | 3108 | 2244 | 463 | 401 |
|  |  | 4 | 65 | 257 | 3602 | 2682 | 639 | 281 |
|  |  | 5 | 65 | 250 | 3600 | 2728 | 611 | 261 |

**Table S3.** Scalability verification results for ECG-GraphNet with excluding both ends.

| **Dataset** | **Total** | ***N*** | ***S*** | ***V*** | **Exact match accuracy***  **(%)** |
| --- | --- | --- | --- | --- | --- |
|  | **Macro F1 (%)** | **F1 (%)** | | |  |
| **8-beat training set** | 87.99 (±2.13) | 97.37 (±1.13) | 80.46 (±5.92) | 86.14 (±2.42) | 73.95 (±5.58) |
| **Pattern test set** | 85.40 | 85.90 | 85.95 | 84.36 | 57.84 |
| **Size test set** | 87.55 | 95.90 | 84.40 | 82.35 | 56.87 |
| **Control test set** | 89.27 | 97.35 | 87.05 | 83.41 | 76.97 |

*Exact match accuracy is the accuracy with which all beats in the graph are completely matched.

**REFERENCES**

1. Das MK, Ari S: ECG Beats Classification Using Mixture of Features. Int Sch Res Notices [Internet] John Wiley & Sons, Ltd, 2014 [cited 2024 Nov 13]; 2014:178436. Available from: https://onlinelibrary.wiley.com/doi/full/10.1155/2014/178436

2. Aziz S, Ahmed S, Alouini MS: ECG-based machine-learning algorithms for heartbeat classification. Scientific Reports 2021 11:1 [Internet] Nature Publishing Group, 2021 [cited 2024 Nov 13]; 11:1–14. Available from: https://www.nature.com/articles/s41598-021-97118-5

3. Sellami A, Hwang H: A robust deep convolutional neural network with batch-weighted loss for heartbeat classification. Expert Syst Appl Pergamon, 2019; 122:75–84.

4. Alamatsaz N, Tabatabaei L, Yazdchi M, Payan H, Alamatsaz N, Nasimi F: A lightweight hybrid CNN-LSTM explainable model for ECG-based arrhythmia detection. Biomed Signal Process Control Elsevier, 2024; 90:105884.

5. Guo L, Sim G, Matuszewski B: Inter-patient ECG classification with convolutional and recurrent neural networks. Biocybern Biomed Eng Elsevier, 2019; 39:868–879.

6. Oh SL, Ng EYK, Tan RS, Acharya UR: Automated diagnosis of arrhythmia using combination of CNN and LSTM techniques with variable length heart beats. Comput Biol Med Pergamon, 2018; 102:278–287.

7. Li Y, Qian R, Li K: Inter-patient arrhythmia classification with improved deep residual convolutional neural network. Comput Methods Programs Biomed Elsevier, 2022; 214:106582.

8. Li Q, Liu C, Li Q, et al.: Ventricular ectopic beat detection using a wavelet transform and a convolutional neural network. Physiol Meas [Internet] IOP Publishing, 2019 [cited 2024 Nov 8]; 40:055002. Available from: https://iopscience.iop.org/article/10.1088/1361-6579/ab17f0

9. Roy M, Majumder S, Halder A, Biswas U: ECG-NET: A deep LSTM autoencoder for detecting anomalous ECG. Eng Appl Artif Intell Pergamon, 2023; 124:106484.

10. Ebrahimi Z, Loni M, Daneshtalab M, Gharehbaghi A: A review on deep learning methods for ECG arrhythmia classification. Expert Systems with Applications: X Elsevier, 2020; 7:100033.

11. Boda S, Mahadevappa M, Kumar Dutta P: An automated patient-specific ECG beat classification using LSTM-based recurrent neural networks. Biomed Signal Process Control Elsevier, 2023; 84:104756.

12. Ansari Y, Mourad O, Qaraqe K, Serpedin E: Deep learning for ECG Arrhythmia detection and classification: an overview of progress for period 2017–2023. Front Physiol Frontiers Media SA, 2023; 14:1246746.

13. Shah HA, Saeed F, Diyan M, Almujally NA, Kang JM: ECG-TransCovNet: A hybrid transformer model for accurate arrhythmia detection using Electrocardiogram signals. CAAI Trans Intell Technol [Internet] John Wiley & Sons, Ltd, 2024 [cited 2024 Nov 13]; . Available from: https://onlinelibrary.wiley.com/doi/full/10.1049/cit2.12293

14. Zhao Z: Transforming ECG Diagnosis:An In-depth Review of Transformer-based DeepLearning Models in Cardiovascular Disease Detection. 2023 [cited 2024 Nov 12]; . Available from: https://arxiv.org/abs/2306.01249v1

15. Hu R, Chen J, Zhou L: A transformer-based deep neural network for arrhythmia detection using continuous ECG signals. Comput Biol Med Pergamon, 2022; 144:105325.

16. Ji C, Wang L, Qin J, Liu L, Han Y, Wang Z: MSGformer: A multi-scale grid transformer network for 12-lead ECG arrhythmia detection. Biomed Signal Process Control Elsevier, 2024; 87:105499.

17. Che C, Zhang P, Zhu M, Qu Y, Jin B: Constrained transformer network for ECG signal processing and arrhythmia classification. BMC Med Inform Decis Mak [Internet] BioMed Central Ltd, 2021 [cited 2024 Nov 13]; 21:1–13. Available from: https://link.springer.com/articles/10.1186/s12911-021-01546-2

18. Hossain MB, Bashar SK, Walkey AJ, McManus DD, Chon KH: An Accurate QRS Complex and P Wave Detection in ECG Signals Using Complete Ensemble Empirical Mode Decomposition with Adaptive Noise Approach. IEEE Access Institute of Electrical and Electronics Engineers Inc., 2019; 7:128869–128880.

19. Costa R, Winkert T, Manhães A, Teixeira JP: QRS Peaks, P and T Waves Identification in ECG. Procedia Comput Sci Elsevier, 2021; 181:957–964.

20. Tan KF, Chan KL, Choi K: Detection of the QRS complex, P wave and T wave in electrocardiogram. IEE Conference Publication IEE, 2000; :41–47.

21. McGuirk SM, Muir WW: Diagnosis and Treatment of Cardiac Arrhythmias. Veterinary Clinics of North America: Equine Practice Elsevier, 1985; 1:353–370.

22. Chatterjee HK, Gupta R, Mitra M: A statistical approach for determination of time plane features from digitized ECG. Comput Biol Med Pergamon, 2011; 41:278–284.

23. Petrenas A, Marozas V, Sörnmo L: Low-complexity detection of atrial fibrillation in continuous long-term monitoring. Comput Biol Med Pergamon, 2015; 65:184–191.

24. De Chazal P, O’Dwyer M, Reilly RB: Automatic classification of heartbeats using ECG morphology and heartbeat interval features. IEEE Trans Biomed Eng 2004; 51:1196–1206.

25. Martínez A, Alcaraz R, Rieta JJ: Ventricular activity morphological characterization: Ectopic beats removal in long term atrial fibrillation recordings. Comput Methods Programs Biomed Elsevier, 2013; 109:283–292.

26. B S, R C, BJ D, et al.: AHA/ACCF/HRS recommendations for the standardization and interpretation of the electrocardiogram: part III: intraventricular conduction disturbances: a scientific statement from the American Heart Association Electrocardiography and Arrhythmias Committee, Council on Clinical Cardiology; the American College of Cardiology Foundation; and the Heart Rhythm Society. Endorsed by the International Society for Computerized Electrocardiology. J Am Coll Cardiol [Internet] J Am Coll Cardiol, 2009 [cited 2025 Feb 17]; 53:976–981. Available from: https://pubmed.ncbi.nlm.nih.gov/19281930/

27. Hannun AY, Rajpurkar P, Haghpanahi M, et al.: Cardiologist-level arrhythmia detection and classification in ambulatory electrocardiograms using a deep neural network. Nature Medicine 2019 25:1 [Internet] Nature Publishing Group, 2019 [cited 2024 Nov 12]; 25:65–69. Available from: https://www.nature.com/articles/s41591-018-0268-3

28. He K, Zhang X, Ren S, Sun J: Deep residual learning for image recognition. Proceedings of the IEEE Computer Society Conference on Computer Vision and Pattern Recognition IEEE Computer Society, 2016; 2016-December:770–778.
